# Clinical Significance of MYT1L Gene Polymorphisms in Chinese Patients with Gastric Cancer

**DOI:** 10.1371/journal.pone.0071979

**Published:** 2013-08-28

**Authors:** Yangmei Zhang, Haixia Zhu, Xunlei Zhang, Dongying Gu, Xichang Zhou, Meilin Wang, Chunxiang Cao, Xiaojing Zhang, Xiaomin Wu, Weida Gong, Yongfei Tang, Jianwei Zhou, Cuiju Tang, Zhengdong Zhang, Jinfei Chen

**Affiliations:** 1 Department of Oncology, Nanjing First Hospital, Nanjing Medical University, Nanjing, China; 2 Department of Oncology, Xuzhou Central Hospital, Affiliated Xuzhou Hospital, College of Medicine, Southeast University, Xuzhou, Nanjing; 3 Department of Environmental Genomics, Jiangsu Key Laboratory of Cancer Biomarkers, Prevention and Treatment, Cancer Center, Nanjing Medical University, Nanjing, China; 4 Department of Genetic Toxicology, the Key Laboratory of Modern Toxicology of Ministry of Education, School of Public Health, Nanjing Medical University, Nanjing, China; 5 Department of Imaging and Nuclear Medicine, Affiliated Hospital of Qinghai University, Medical College of Qinghai University, XiNing, China; 6 Department of Surgery, Yixing Cancer Hospital, Yixing, China; 7 Department of Surgery, Yixing People's Hospital, Yixing, China; 8 Department of Molecular Cell Biology and Toxicology, School of Public Health, Cancer Center, Nanjing Medical University, Nanjing, China; Sapporo Medical University, Japan

## Abstract

**Background:**

Myelin transcription factor 1 (MYT1) and its homologue MYT1-like (MYT1L) are the two main members of MYT/NZF family transcription factors, which are highly related, share a high degree of identity and show similar regulatory functions in neural development. There are evidences from several cytology experiments showing that MYT1 is associated with carcinoma.

**Methodology/Principal Findings:**

In the present study, we genotyped 944 surgically resected gastric cancer patients by the SNaPshot method to explore the association of MYT1L rs17039396 polymorphism with survival of gastric cancer in a Chinese population. We found that cardia cancer patients carrying MYT1L rs17039396 GG genotype survived for a significantly shorter time than those carrying the GA genotype. This significance was enhanced in the dominant model (GG vs. GA/AA, log-rank *P* = 0.001), suggesting a potential protect role of the variant A allele. Multivariate Cox regression analyses showed that the AG/GG genotypes were associated with a significantly decreased risk of death from gastric cancer (adjusted hazard ratio (HR) = 0.57, 95% confidence interval (CI) = 0.40–0.81). Stratification analyses further showed that such protective effect was statistically significant in subgroups of patients with tumor size ≤5 cm (adjusted HR = 0.34, 95%CI = 0.19–0.64), well-moderate gastric cancer (adjusted HR = 0.59, 95%CI = 0.35–0.98), no lymph-node metastasis (adjusted HR = 0.49, 95%CI = 0.31–0.76), no distant metastasis (adjusted HR = 0.59, 95%CI = 0.41–0.84).

**Conclusions/Significance:**

In conclusion, these data represents the first demonstration that MYT1L rs17039396 variants could indentified as a favorable prognostic indicator for gastric cancer, particularly among the cardia gastric cancer. Further validation in other larger studies with different ethnic populations and functional evaluations are needed.

## Introduction

Gastric cancer (GC) represents the fourth most common cancer and the second leading cause of cancer-related mortality worldwide [1]. The highest incidence and mortality rates are observed in East Asia, predominantly in China [1]. Remarkable improvements have been made to date in comprehensive treatment strategies of combined surgery, chemotherapy, radiotherapy, and targeted therapy, but GC patients still have a poor prognosis, with 5-year overall survival rates of 30% [2]. Although TNM classification has been widely considered as the best available clinical measure of tumor aggression and prognosis, obvious differences exit even among patients in the same stage [3]. Moreover, multiple genetic and epigenetic alterations are implicated in the multistep process of human stomach carcinogenesis and development [4]. Therefore, discovery of readily accessible molecular markers and their application in incorporated with traditional cancer diagnosis, staging and prognosis could to a large extent be helpful for the improvement of early diagnosis, screening of high-risk individuals, as well as patient care [5]. In recent years, increased studies had focused on the detection of genetic variants that could play roles in the development and progression of gastric cancer [6].

Zinc finger (ZnF) gene family constitute one of the largest gene families, accounting for about 3% of the genes of the human genome [7], and serve important functions in a diverse array of developmental events and cellular processes, such as control of cellular proliferation, differentiation, development and death [8]. Myelin transcription factor 1 (MYT1, or neural zinc finger 2 (NZF2)) and its homologue MYT1-like (MYT1L, or NZF1) are the two main members of MYT/NZF family transcription factors, each of which contains six copies of a ZnF with a C_2_HC consensus sequence. They are highly related and display a high degree of identity (91% for the finger regions and 62% at the protein level) [9], [10]. Moreover, both proteins recruit the same histone deacetylases to regulate neural transcription via their interaction with Sin3B [11], pointing to similar regulatory functions in neurogenesis. In order to elucidate the radioimmunotherapy molecular mechanisms of the treatment of gastric cancer cells expressing d9-E-cadherin with ^213^Bi-d9MAb, Seidl et al. [12] quantified 380 gene expression of ^213^Bi-treated tumor cells and found that ^213^Bi-induced cell death was initiated by G2 arrest and up-regulation of several genes, including MYT1. In addition, a cytology study revealed that the c-Jun N-terminal kinase -mediated phosphorylation of MYT1, accompanying with MYT1 overexpression, played an important role in UVA-induced apoptosis and the suppression of skin carcinogenesis [13]. Although MYT1L itself has not previously been reported to associated with carcinoma, it is highly homologous to MYT1 [9], [10] and the loss of MYT1 function may be compensated by MYT1L activity [14]. So we hypothesize that MYT1L gene may also be linked to gastric cancer. Yang et al. analyzed 444,044 germline genetic single nucleotide polymorphisms (SNPs) in an ethnically diverse group of 2,534 children with acute lymphoblastic leukemia and provided significant evidences that a locus at 2p25.3 (rs17039396) in MYT1L gene exhibits the strongest association with relapse of disease [15]. Therefore, we conduct this study to examine whether MYT1L rs17039396 polymorphism has potential significance as molecular prognostic markers for gastric cancer, which will help further define sub-populations who are at higher risk of poor disease and consequently require more aggressive treatment and more rigorous postoperative follow up.

## Materials and Methods

### Study population

The whole study was approved by the Institutional Review Board of Nanjing Medical University (Nanjing, China), and each of the patients signed an informed consent on the use of clinical specimens for gene polymorphisms analyses. In this retrospective study, 944 patients with histopathologically confirmed gastric cancer who had received surgical resection between January 1999 and December 2006 were recruited from Yixing People's Hospital, (Yixing, Jiangsu Province, China). None have received prior radiotherapy or chemotherapy before surgery and not all of them have received adjuvant chemotherapy. 944 Formalin-Fixed and Parrffin-Embedded samples were obtained from the department of pathology of this hospital. The end point was overall survival (OS), which was calculated from the date of surgery until death or the last follow-up in March 2009. Death dates were confirmed by review of death certificates of inpatient and outpatient records or obtained through follow-up telephone calls. Patients still alive were censored. The maximum survival time was 119.0 months and the median survival time was 70.0 months. The demographic features and clinicopathological variables were summarized in Table 1, which were obtained from the medical records of the patients. The TNM stage classification was evaluated according to the criteria of the 7th edition of the American Joint Committee on Cancer (AJCC).

**Table 1:** Association between clinicopathological features and survival of gastric cancer.

Variables	Patients, n = 909	Deaths, n = 421	MST (months)	Log-rank p	HR (95% CI)
Age (years)					
≤60	428	197	97	0.431	1.00
>60	481	224	62		1.01 (0.99–1.02)
Sex					
Male	699	322	70	0.564	1.00
Female	210	99	67		1.07 (0.85–1.34)
Tumor size					
≤5 cm	566	238	74	<0.001	1.00
>5 cm	343	183	48		1.43 (1.18–1.73)
Location					
Non-cardia	600	282	67	0.354	1.00
Cardia	309	139	77		0.91 (0.74–1.11)
Histological types					
Intestinal	386	150	77§	<0.001	1.00
Diffuse	519	268	50		1.45 (1.19–1.77)
Others	4	3	11		
Differentiation^a^					
Well to moderate	296	126	77	<0.001	1.00
Poorly	473	230	62		1.15 (0.93–1.43)
Mucinous/signet-ring cell	65	32	62		1.19 (0.81–1.75)
Depth of invasion^b^					
T1	178	58	84§	<0.001	1.00
T2	130	56	78§		1.42 (0.99–2.06)
T3	6	3	70		1.42 (0.44–4.52)
T4	576	292	51		1.82 (1.38–2.42)
Lymph node metastasis^c^					
N0	360	130	80	<0.001	1.00
N1/N2/N3	549	291	47		1.72 (1.41–2.13)
Distant metastasis					
M0	857	391	74	0.015	1.00
M1	52	30	26		1.58 (1.09–2.29)
TNM stage					
I	241	82	83	<0.001	1.00
II	194	78	71		1.24 (0.91–1.69)
III	447	245	39		1.96 (1.52–2.52)
IV	27	16	27		2.40 (1.41–4.11)
Smoking					
Non-smoker	833	389	97	0.399	1.00
Smoker	76	32	65		0.86 (0.60–1.23)
Chemotherapy					
No	298	136	74	0.516	1.00
Yes	611	285	60		1.07 (0.87–1.31)

Abbreviation: MST, median survival time; HR, hazard ratio; CI, confidence interval; AJCC, American Joint Commission on Cancer.

a Partial data were not available, and statistics were based on available data.

b The information about the depth of invasion was not available for two patients; invaded depth of tumor was classified according to the criteria of AJCC 7th.

c Lymph nodes were staged according to tumor-node-metastasis classification of the 7th edition of AJCC in which the number of lymph nodes with a metastasis of 1∼2, 3∼6 and ≥7 were classified as N1, N2 and N3, respectively.

§ Mean survival time was presented when the median survival time could not be measured.

### Genotyping

Genomic DNA was extracted from paraffin-embedded tumor bearing tissue using proteinase K digestion, followed by isopropanol extraction and ethanol precipitation. Genotyping of samples was conducted by multiplex SNaPshot technology using an ABI fluorescence-based assay allelic discrimination method (Applied Biosystems, Foster city, CA, USA), which has been described in detail in previous study [16]. The primers were designed to anneal immediately adjacent to the nucleotide at the mutation site: forward, 5′- TAT TAG TCT GAR TCT GCT GGC CTT TG -3′; reverse, 5′- GAC TTC ACC TCC ACC AGG ACC A-3′. The primers for extension were as follows: 5′-TTT CCT CCA CCA GGA CCA GAT TT-3′. The SNaPshot products were analyzed by using an ABI 3130 genetic analyzer (Applied Biosystems) and the genotypes were determined by GeneMapper Analysis Software version 4 (Applied Biosystems). Genotype analysis was performed by two investigators blinded to the survival end points. Genotyping was validated by sequencing a randomly selected 10% of samples, and the results were 100% concordant. However, 35 cases failed in genotyping because of poor DNA quality, which were excluded in further analysis. Finally, 909 gastric cancer patients were included in the analysis.

### Statistical analysis

Statistical analyses were carried out using SPSS version 16.0 for Windows (SPSS Inc., Chicago, IL, USA). Kaplan-Meier survival curves and the log-rank test were used for survival analysis. Univariate or multivariate Cox proportional hazard models was adopted to estimate the crude hazard ratios (HRs), adjusted HRs and their 95% confidence intervals (CIs), with adjustment for potential confounders. Moreover, Cox stepwise regression analysis was performed to asses the independent impacts of SNP or clinicopathological features on the overall survival after adjusting for other covariates, with a significance level of *P*<0.05 for entering and *P*>0.10 for removing the respective explanatory variables. Hardy-Weinberg equilibrium of alleles at individual loci was assessed by a goodness-of-fit χ^2^ test. All tests were two-sided and *P*<0.05 was considered statistically significant.

## Results

### Associations between clinicopathological variables and overall survival

The final population of this study consisted of 909 patients. The patients characteristics and clinical information are summarized in Table 1. In the follow-up period of 119 months, 421 patients died. There were 699 males (76.9%) and 210 females (23.1%), with the median age of 62 years ranging from 28 to 83 years. Clinicopathological characteristics including tumor size, histological types, differentiation, depth of invasion, lymph node metastasis, distant metastasis and TNM stage were significantly associated with survival time (log-rank *P*<0.05). Specifically, patients with tumor size >5 cm (MST, 48 months) had a 43% significantly higher risk of death (HR = 1.43, 95% CI = 1.18–1.73), compared with those with tumor size ≤5 cm (MST, 74 months), and the diffuse-type gastric cancer patients (MST, 50 months) had a 45% significantly higher risk of death (HR = 1.45, 95% CI = 1.19–1.773), than those intestinal-type patients (MST, 77 months). In addition, as the depth of invasion and TNM stage increased, the risk of death for gastric cancer showed a significant increase in a dose-response manner (log-rank *P*<0.001).

### Associations of MYT1L rs17039396 with clinical outcomes of GC

**Figure 1:**
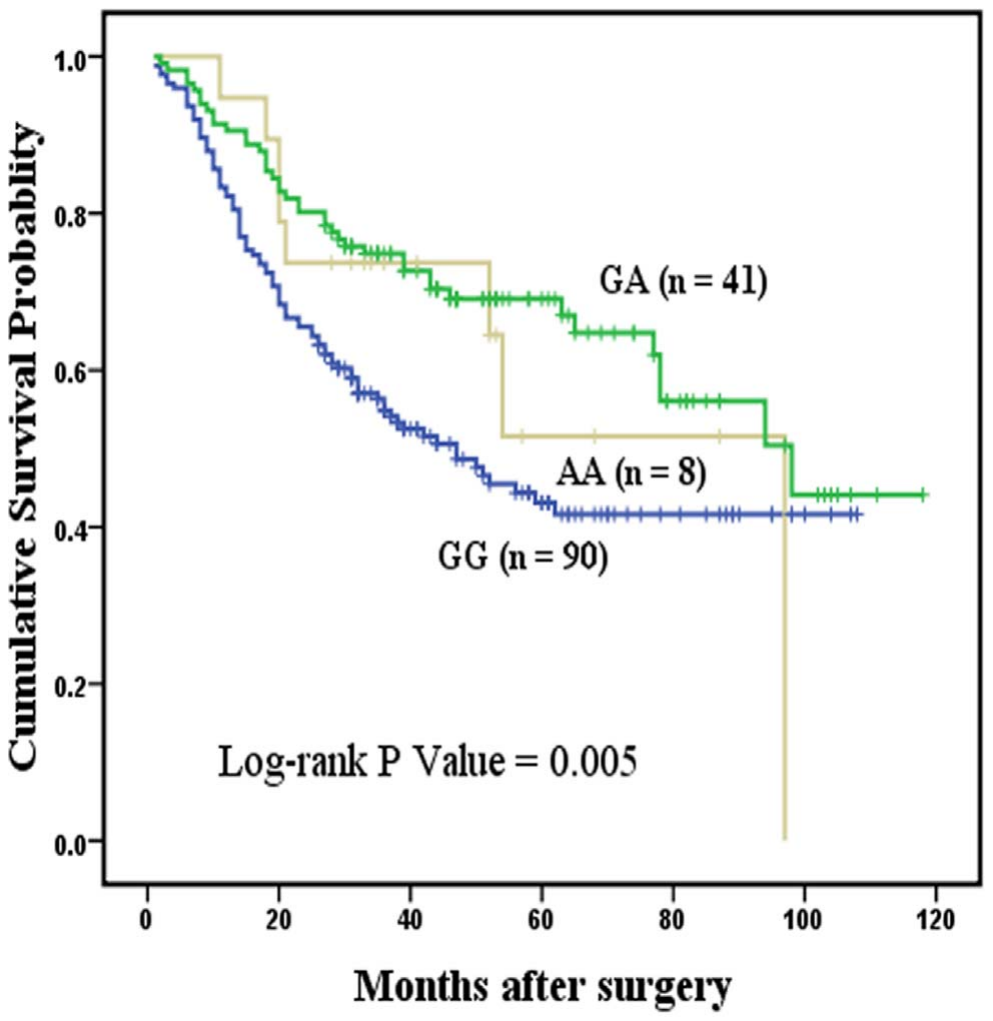
Overall survival curve in relation to MYT1L rs17039396 polymorphism in patients with cardia gastric cancer in overall model. Figure 1 represents the Kaplan-Meier survival curve in relation to the effect of MYT1L variants on overall survival of the patients with cardia gastric cancer in overall model and the *P* value of log-rank test received statistical significance.

**Figure 2:**
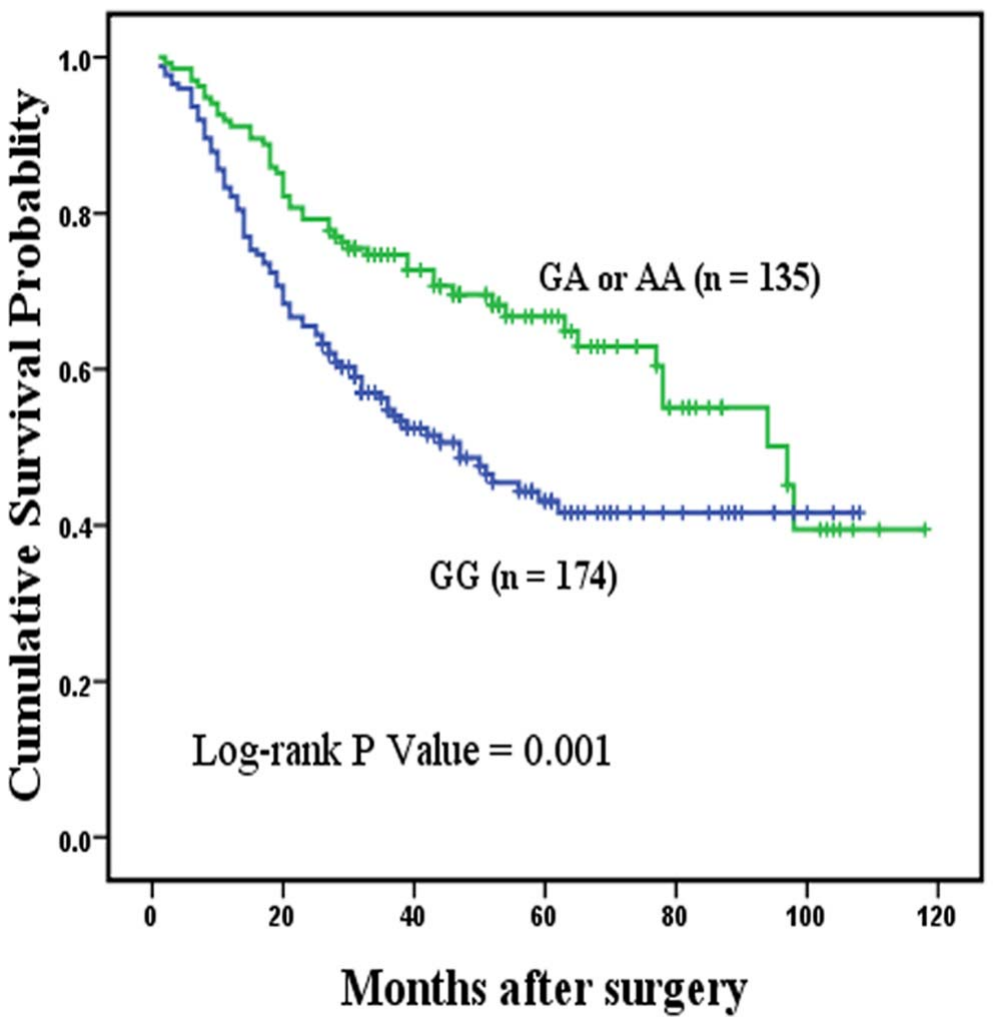
Overall survival curve in relation to MYT1L rs17039396 polymorphism in patients with cardia gastric cancer in dominant model. Figure 2 represents the Kaplan-Meier survival curve in relation to the effect of MYT1L rs17039396 polymorphism on overall survival of the patients with cardia gastric cancer in dominant model. Patients with GA or AA genotypes was at lower risk of death, compared with those with GG homozygotes. *P* value is 0.001, suggesting that MYT1L rs17039396 GA+AA genotypes were associated with better overall survival in 309 patients with cardia gastric cancer.

Among 944 specimens of GC patients, MYT1L rs17039396 was successfully genotyped in 909 specimens. The frequency of each genotypes was 57.0% (518 specimens) for the GG variant, 37.8% (344 specimens) for the GA variant, and 5.2% (47 specimens) for the AA variant. The genotype frequencies of MYT1L rs17039396 in the cases followed HWE (*P* = 0.21). The Kaplan-Meier survival analysis and Cox proportional hazard models were used to assess the prognostic effect of MYT1L rs17039396 on GC patients in different genetic models (Table 2). No significant associations were observed between the MYT1L rs17039396 genotypes and OS of GC patients in any genetic models. We further evaluated the associations by stratified analysis of tumor location and histological types. In the overall model, MYT1L rs17039396 polymorphism was associated with the survival of cardia cancer (log-rank *P* = 0.015, Fig. 1). Survival time of patients with the GA genotype (MST 98 months) was significantly different compared with that of patients with the GG genotype (MST 47 months), who had a 44% higher risk of death (HR = 0.56, 95% CI = 0.39–0.81). In the dominant model, a significantly lower risk of death (HR = 0.57, 95% CI = 0.40–0.81) was found (log-rank *P* = 0.001), as shown in Fig. 2.

**Table 2:** Association between MYT1L rs17039396 polymorphism and overall survival of gastric cancer.

	All patients					
Genetic models	Genotypes	Patients	Deaths	MST (months)	Log-rank *p*	HR (95%CI)^a^
Overall model	GG	518	248	56	0.108	1.00
	GA	344	147	80		0.84 (0.68–1.39)
	AA	47	26	52§		1.21 (0.80–1.81)
Dominant model	GG	518	248	56	0.185	1.00
	GA or AA	391	173	78		0.88 (0.72–1.06)
		Non-cardia cancer				
Overall model	GG	344	158	74	0.134	1.00
	GA	228	106	67		1.01 (0.79–1.29)
	AA	28	18	22§		1.62 (0.99–2.64)
Dominant model	GG	344	158	74	0.546	1.00
	GA or AA	256	124	63		1.07 (0.84–1.35)
		Cardia cancer				
Overall model	GG	174	90	47	0.005	1.00
	GA	116	41	98		0.56 (0.39–0.81)
	AA	19	8	97§		0.67 (0.33–1.38)
Dominant model	GG	174	90	47	0.001	1.00
	GA or AA	135	49	97		0.57 (0.40–0.81)

MST, median survival time; HR, hazard ratio; CI, confidence interval.

a Hazard Ratio (HR) adjusted for age, sex, TNM stage.

§ Mean survival time was presented when the median survival time could not be measured.

### Further stratified analyses and stepwise Cox regression model for survival among cardia cancer

The contribution of MYT1L rs17039396 A allele to the improved survival of cardia gastric cancer patients were further evaluated by stratified analysis of tumor size, histological types, degree of differentiation, depth of invasion, lymph node metastasis, distant metastasis and TNM stage. As a result, in the stratified analysis among cardia gastric cancer, we found that this protect effect was more prominent among subgroups of patients with tumor size ≤5 cm (adjusted HR = 0.34, 95%CI = 0.19–0.64), well-moderate gastric cancer (adjusted HR = 0.59, 95%CI = 0.35–0.98), no lymph-node metastasis (adjusted HR = 0.49, 95%CI = 0.31–0.76), no distant metastasis (adjusted HR = 0.59, 95%CI = 0.41–0.84) (Table 3). Cox stepwise regression analysis was conducted to evaluate the independent effect of clinicopathological variables and rs17039396 SNP on the OS of the patients with cardia gastric cancer. As shown in Table 4, two variables (TNM stage and MYT1L rs17039396) were included in the regression model by stepwise selection of the covariant variables and rs17039396 SNP was shown to be an independent protective factor for cardia cancer with a 44% decreased risk (HR = 0.56, 95%CI = 0.39–0.79, *P* = 0.001).

**Table 3:** Stratified analysis of MYT1L rs17039396 polymorphism among cardia cancer patients.

Variables	Genotypes (deaths/patients)		HR (95% CI)^a^	*P*
	GG	GA/AA		
Total	90/174	49/135	0.57 (0.40–0.81)	0.002
Tumor size				
≤5 cm	53/115	31/89	0.34 (0.19–0.64)	0.001
>5 cm	37/59	18/46	0.67 (0.43–1.05)	0.082
Histological types				
Intestinal	42/84	26/72	0.61 (0.38–1.01)	0.053
Diffuse	48/90	26/72	0.52 (0.32–1.07)	0.072
Differentiation				
Well to moderate	42/75	23/57	0.59 (0.35–0.98)	0.042
Poorly	37/77	23/61	0.67 (0.33–1.13)	0.129
Mucinous/signet-ring cell	5/8	1/6	0.13 (0.01–1.39)	0.092
Depth of invasion				
T1	4/19	5/20	1.22 (0.30–4.99)	0.778
T2	13/28	7/20	0.70 (0.27–1.79)	0.455
T3	0/1	0/0	—	
T4	72/125	36/92	0.52 (0.35–1.18)	0.062
Lymph node metastasis				
N0	30/69	17/56	0.49 (0.31–0.76)	0.002
N1/N2/N3	60/105	32/79	0.64 (0.36–1.17)	0.148
Distant metastasis				
M0	65/166	48/131	0.59 (0.41–0.84)	0.004
M1	5/8	1/4	—	
TNM stage				
I	14/37	8/32	0.55 (0.23–1.32)	0.181
II	21/45	12/35	0.75 (0.37–1.54)	0.437
III	52/88	28/67	0.46 (0.28–1.05)	0.202
IV	3/4	1/1	1.47 (0.03–66.46)	0.842

HR, hazard ratio; CI, confidence interval.

a Hazard Ratio (HR) adjusted for age, sex.

**Table 4:** Stepwise Cox regression analysis on the survival of cardia cancer.

Variables	β	SE	HR	95%CI	*P* value
Age^a^	0.006	0.005	1.01	(0.99–1.02)	0.235
Sex	0.066	0.115	1.07	(0.85–1.34)	0.567
TNM stage	0.388	0.110	1.47	(1.19–1.83)	<0.001
rs17039396 (GG *vs* GA/AA)	−0.582	0.179	0.56	(0.39–0.79)	0.001

β, regression coefficient; SE, standard error; HR, hazard ratio; CI, confidence interval.

a Age was included as a continuous variable in the Cox stepwise regression analysis.

## Discussion

In the present study, we investigated the effect of the MYT1L rs17039396 SNP on the progression and survival of GC. Our results indicated that the heterozygote genotype (GA) had a significantly higher survival rate than homozygote genotype (GG), and the association was also observed when analyzing the dominant model (GA/AA *vs* GG), suggesting that the MYT1L rs17039396 A allele may be associated with survival of GC.

In our results, MYT1L rs17039396 was significantly correlated with improved survival of cardia carcinoma but not noncardia carcinoma of the stomach. There is recently increasing evidence that the cardia type of gastric cancer has different characteristics from the noncardia type in terms of aetiology, pathology, carcinogenesis, biological behavior, prognosis and even genetic background. For example, Kamangar et.al reported that H. pylori infection was a strong risk factor for non-cardia gastric cancer but was inversely associated with the risk of gastric cardia cancer [17]. Compared with the non-cardia gastric cancer, gastric cardia cancer is associated with reflux symptoms, predominance in white males and a greater frequency of differentiated-type tumors [18]. Furthermore, a greater tendency towards poorly differentiated histology, lymph node metastasis, advanced pathologic TNM stage, and a poor prognosis were described as characteristics of cardia carcinoma [19]. Therefore, it is rational to consider cardia carcinoma as a specific category of GC. It could be said that the indiscriminate combination of the two subtypes of GC may mask or produce underestimation of the strength of the authentic associations. In the stratified analyses, when confined to the patients with some special clinicopathological features such as tumor size ≤5 cm, well-moderate gastric cancer, no lymph-node metastasis, no distant metastasis, the survival time for the subjects carrying GA or AA genotypes was longer than those for GG genotypes, indicating that the abovementioned prognostic factors may have a combined effect with rs17039396 on the superior OS of cardia gastric cancer.

The MYT1L gene (MIM:613084) maps to chromosome 2p25.3 with 542161 bp in length, comprising twenty-five exons (http://www.ncbi.nlm.nih.gov/GENE/). Exon 1 to exon 5 and the distal part of exon 25 are untranslated regions, while the other 19 exons and the proximal part of exon 25 are coding regions. Wang et al. [20] found that rs3748989 in exon 9 of MYT1L gene conferred a predisposition to major depressive disorder. A case–control study with a relatively large sample size showed that rs17039584 located near 5′ untranslated regions and rs10190125 in intron 1 of MYT1L gene were significantly associated with Schizophrenia [21]. Our study revealed a significant correlation of rs17039396 located at intron 3 with cardia gastric cancer. Although the roles of these SNPs in MYT1L gene expression and their precise functional and biological significances have been largely unknown, it is not difficult to speculate that antonymous mutations occurring in exons in coding regions could lead to substitutions of amine acids, change the structure of coding-proteins, and subsequently affect their biological functions. Whereas, what is the mechanism of action of intronic polymorphic variants? It has increasingly become apparent that intronic germline variations might involve alterations of gene regulation and transcript processing, or produce splicing variants [22]–[24]. Recently, a T to G change at the 309^th^ nucleotide in the first intron of the MDM2 gene (SNP309) has been found and shown to increase the affinity of the transcriptional activator Sp1, resulting in higher levels of MDM2 RNA and protein and the subsequent attenuation of the p53 pathway [25]. Subsequently, a case-control study including 438 controls and 410 patients with sporadic gastric carcinoma provided evidence supporting the association of MDM2 SNP309 with both an increased susceptibility to gastric carcinoma and poor prognosis [26]. Furthermore, Narla et al. [27] reported that a relatively common intronic polymorphism of KLF6 gene enhances alternative splicing and is associated with increased prostate cancer risk. Given above-mentioned evidences, it is reasonable to presume that MYT1L intronic polymorphisms might exert their functions through alternative gene expression or splicing variants that permit the generation of protein isoforms having different biology functions. To our knowledge, there have been no published attempts to characterize the functional implications of the MYT1L intron polymorphisms. Hence, the clear molecular mechanisms by which genetic variants exert their biological implications warrants further experimental investigation.

The MYT1L protein plays a crucial role in CNS development, so it is rational to presume that changes in its expression level or function resulted from genetic variations could lead to psychiatric disorders [20]–[21]. However, exactly how this protein affect the susceptibility, progression or prognosis of tumor is far from clear. The MYT1L protein is a member of the myelin transcription factor 1 family that modulates proliferation and differentiation of oligodendrocytes by controlling the transcriptional activity of downstream genes involved in lineage specification [28]. In addition, Riley et al. [29] hypothesized that MYT1L regulates ZNF804A gene expression in schizophrenia patients. Based on these evidences, we hypothesized that genetic variation in MYT1L gene might affects its function to regulate expression levels of sets of tumor-related genes, and consequently involves in carcinogenesis. Another plausible explanation of its association with gastric cancer lies in controlling cell-cycle progression. Except for the transcriptional activity, MYT1 also functions as a cell cycle-regulated kinase. Dai et al. [30] indicated that the upregulation of Myt1 and Wee1 induced the phosphorylation of Cdc2 leading to G2/M arrest in normal cell line. The p53 tumor suppressor protein plays a crucial role in tumorigenesis and prevents the proliferation of cancer-prone cells primarily by controlling cell-cycle progression and apoptosis. Passer et al. [31] found that up-regulated expression of TSAP6, transcriptionally activated by p53, could augment MYT1 activity, resulting in cell-cycle delay and the suppression of growth of cancer-prone cells. Furthermore, a more recent study investigating the functions of MYT1 in checkpoint recovery followed by DNA damage revealed that downregulation of MYT1 potentiated with DNA damage to inhibit tumor cell growth in tumor xenograft mice models, implicating MYT1 as a potential target for anti-cancer therapies [32]. These data highlight the contribution of MYT1 protein to regulation of cell-cycle progression and implicate it as a potential target for anti-cancer therapies. Although MYT1L has not be reported to function to modulate the cell-cycle progression, it is highly homologous to MYT1 [9]–[10] and the loss of MYT1 function may be compensated by MYT1L activity [14]. So it is plausible that MYT1L protein may have a similar role in regulation of cell-cycle process and their changes stemming from genetic variation may be involved in tumorgentic process. Taken together, the presented findings of the potential involvement of MYT1L gene in antitumorigenesis prompts us to further characterize its structure, biological function, and interaction with other partners by in vitro or vivo studies.

Some limitations of the present study should be addressed. First, only one SNP in MYT1L is evaluated, and it is possible that some other important SNPs are neglected or the observed associations may be due to other polymorphisms in linkage disequilibrium with the rs17039396 polymorphism. Second, Helicobacter pylori, as a known crucial factor in gastric carcinogenesis, was not considered due to the lack of related follow-up information. Finally, for validation of the genotype–phenotype relationship, further investigation is underway to clarify the association between rs17039396 polymorphism and expression levels of MYT1L protein in gastric cancer tissues and will be reported separately.

In conclution, our results represent the first demonstration that MYT1L rs17039396 SNP may be associated with the prognosis of cardia cancer patients. The survival of patients in the dominant model was significantly better than the survival in the overall model, suggesting that the mutant allele may serve as a suitable marker for predicting the survival of cardia cancer patients, especially in a Chinese population. Consequently, testing for MYT1L rs17039396 SNP, combined with other traditional prognostic factors may significantly help distinguish a subgroup of patients with poor prognosis, thereby helping to refine therapeutic decisions in the treatment of gastric cancer.
